# Giant Right Coronary Artery Aneurysm in a Patient With Multiple Coronary Artery Aneurysmatic Dilatations

**DOI:** 10.7759/cureus.51390

**Published:** 2023-12-31

**Authors:** Izatullah Jalalzai, Yasin Kilic, Ebubekir Sönmez, Furkan Çelik, Bilgehan Erkut

**Affiliations:** 1 Cardiovascular Surgery, Ataturk University Hospital, Erzurum, TUR

**Keywords:** cabg surgery, coronary artery bypass grafting(cabg), cardiopulmonary bypass, coronary artery thrombosis, left main coronary artery aneurysm, aneurysm formation

## Abstract

One kind of coronary artery disease that is uncommon is coronary artery aneurysm (CAA). According to angiographic reports, the incidence of coronary artery aneurysms ranges from 1.5% to 4.9%, with a higher frequency in men. A patient with both coronary heart disease and an aneurysm in the right coronary artery (RCA) underwent a successful simultaneous coronary bypass together with an aneurysmal reconstruction procedure.

## Introduction

According to published definitions, a coronary artery aneurysm (CAA) is characterized by a connected blood vessel's diameter that is 1.5 times larger than the diameter of normal coronary arteries. They can be categorized as fusiform or saccular based on their morphology [1, 2]. Although there are numerous acquired causes (trauma, infection, vasculitis, Kawasaki disease, catheterization, surgery, spontaneous dissection, metastatic tumors, etc.), it can also occur congenitally. A CAA may be caused, in part, by atherosclerosis [2, 3]. Although it usually has no symptoms, it can result in myocardial infarction (MI) and clinical angina.

## Case presentation

A male patient, aged 56, who did not have any documented cardiac disease, presented at the cardiology clinic with the following symptoms: retrosternal chest and left arm pain of sudden onset, chest tightness, and shortness of breath. These symptoms had been present for approximately three weeks. In the absence of notable alterations in the electrocardiogram (ECG), the patient had troponin I levels at 1,613 pg/mL. His initial diagnosis was a non-ST elevation MI, which led to his admission to the cardiology department.

**Figure 1:**
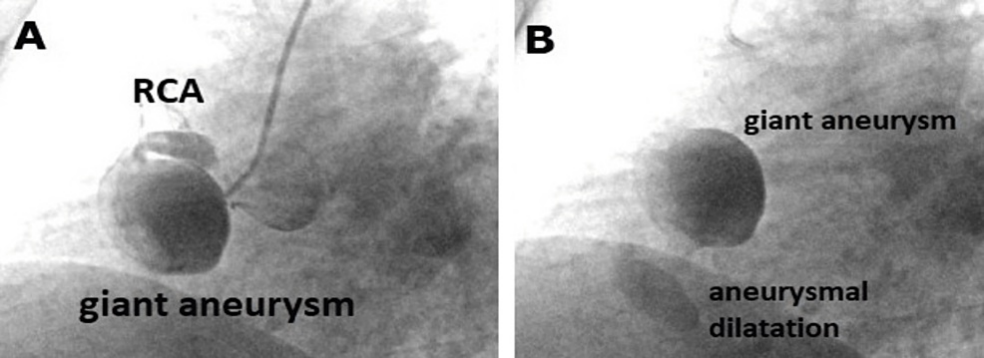
Preoperative angiography Figure 1A: angiographic visualization of the right coronary artery (RCA) and a giant aneurysm; Figure 1B: coronary angiography showing two coronary artery aneurysms together.

In his physical examination, his blood pressure was 110/80 mmHg with a heart rate of 88 pulses per minute. A comprehensive evaluation of the cardiovascular and respiratory systems did not reveal any abnormal or pathological findings. A chest radiograph showed a high cardiothoracic ratio with a normal electrocardiogram (ECG). A catheter angiography was planned. Forty percent stenosis, coronary dilatation in the proximal left anterior descending (LAD) artery, and ectasia in the circumflex artery were found. In addition, two aneurysms measuring 52 × 45 mm (Figure 1A) and 22 × 20 mm (Figure 1B) were found in the right coronary artery (RCA), respectively.

One of the aneurysms originated from the middle and proximal segments of the RCA and was partially thrombosed. Surgery was planned, and the patient was referred to our cardiovascular surgery clinic for an operation.

**Figure 2:**
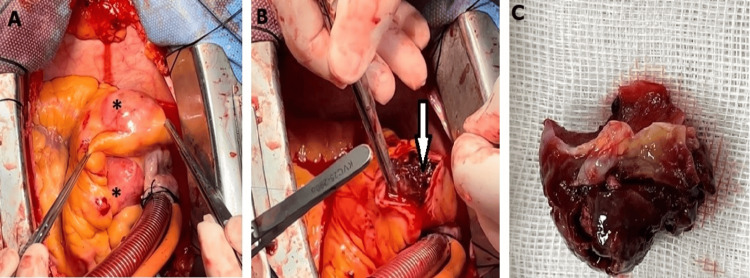
Intraoperative interpretation Figure 2A: double aneurysms in the right coronary artery (asterisk); Figure 2B: thrombosed coronary artery aneurysm (white arrow); Figure 2C: removed thrombus from coronary artery aneurysm

After routine laboratory tests and anesthetic premedication, the patient was operated on for coronary artery bypass and CAA interventions. After general anesthesia, a median sternotomy was performed, and the pericardium was opened. Firstly, aneurysm sacs, one of which was very large, were seen in the RCA area (approximately 2 x 3 cm and 5 x 4 cm in size) (Figure 2A).

Cardiopulmonary bypass was initiated via aorto-caval cannulation. After cross-clamping and cardioplegic administration, the aneurysm sacs were individually explored, and the dense thrombus was removed (Figures 2B-2C).

**Figure 3:**
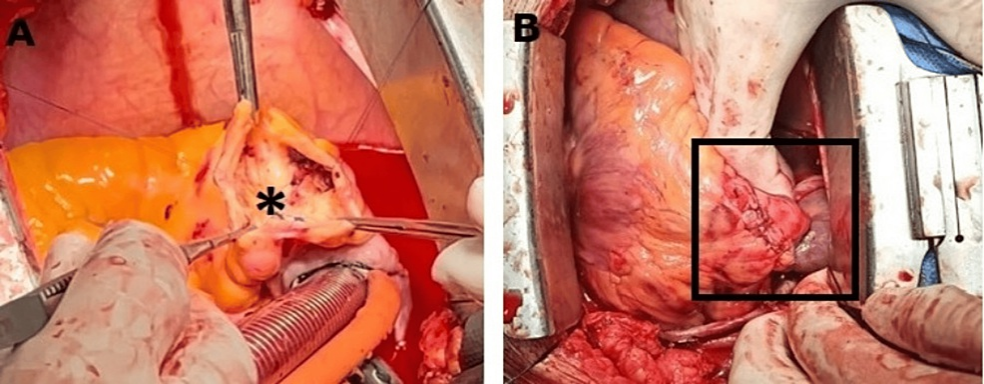
Postoperative images Figure 3A: inner view of aneurysm lumen after removal of thrombus and ligation of proximal and distal coronary ostium (asterisk); Figure 3B: repair of the defected aneurysm wall (black frame).

Both distal and proximal coronary arteries were sutured, and the sacs were closed (Figures 3A-3B).

A vein graft was used to bypass the distal RCA. In addition, the left internal mammary artery was anastomosed to the LAD artery, and another vein graft was used to bypass the circumflex (Cx) artery. The patient emerged from cardiopulmonary bypass without incident and was transferred to the intensive care unit. The patient was discharged on the eighth day after the surgical procedure without any complications.

## Discussion

The prevalence of CAA varies between 0.3% and 5.3%, with an average prevalence of 1.65% [3, 4]. Males have a greater incidence of CAAs [4]. The RCA (40.4%) is most commonly affected, followed by the LAD artery (32.3%) and the left Cx artery (23.4%) [5]. Typically, CAAs are asymptomatic lesions. Usually, patients who have heart failure or ischemia with symptoms refer to the cardiology clinic [6, 7]. In the case we present, the patient exhibited chest pain that typically extends to the left arm and was experiencing difficulty breathing.

There is still a lack of clarity regarding the cause, but the evidence is growing. Heart conditions that can lead to coronary aneurysms may include atherosclerosis, syphilis, Kawasaki disease, Ehlers-Danlos syndrome, trauma, iatrogenic herbicide poisoning that causes chronic nitric oxide stimulation after surgery, trauma, and congenital malformations. In our case, the etiology of the condition was assumed to be atherosclerosis, as determined by the analysis of the pathological findings. Coronary artery aneurysms have the potential to remain asymptomatic or result in significant morbidity and mortality due to complications such as distal embolization, fistula formation, thrombus formation, dissection, or rupture [6, 8]. In the present case, the patient had MI, which was probably attributed to distal micro-embolization. Whatever the underlying process leading to CAAs, they do not appear to be benign. This has the potential to trigger acute MI characterized by spontaneous dissection in the absence of stenosis, spasm, or thrombosis.

A CAA's prognosis in the setting of ischemic heart disease is severity-dependent. The presence of stenosis, the aneurysm's size, and the risk of rupture are surgical indications for CAAs. Furthermore, the magnitude of the fistula and its presence in a minimum of one chamber of the heart are further factors to be taken into account.

## Conclusions

The occurrence of CAA, although infrequent, is linked to a significant fatality rate in the context of ischemic heart disease. The appropriate course of treatment for this condition is contingent upon the specific characteristics of the aneurysm. Rupture or thromboembolic blockage is a significant risk factor, thereby necessitating surgical intervention for treatment. While there are a variety of surgical techniques, the fundamental approach to surgical intervention involves either the removal of the aneurysm or its repair while guaranteeing the uninterrupted flow of coronary blood.
